# A Critical Review of the Evidence That Metformin Is a Putative Anti-Aging Drug That Enhances Healthspan and Extends Lifespan

**DOI:** 10.3389/fendo.2021.718942

**Published:** 2021-08-05

**Authors:** Ibrahim Mohammed, Morley D. Hollenberg, Hong Ding, Chris R. Triggle

**Affiliations:** ^1^Department of Medical Education, Weill Cornell Medicine-Qatar, Al-Rayyan, Qatar; ^2^Inflammation Research Network and Snyder Institute for Chronic Diseases, Department of Physiology & Pharmacology, University of Calgary Cumming School of Medicine, Calgary, AB, Canada; ^3^Department of Medicine, University of Calgary Cumming School of Medicine, Calgary, AB, Canada; ^4^Departments of Medical Education and Pharmacology, Weill Cornell Medicine-Qatar, Al-Rayyan, Qatar

**Keywords:** metformin, aging, healthspan and lifespan, AMP-Kinase, calorie restriction mimetic, diabetes, cardiovascular and neurodegenerative diseases, cancer

## Abstract

The numerous beneficial health outcomes associated with the use of metformin to treat patients with type 2 diabetes (T2DM), together with data from pre-clinical studies in animals including the nematode, C. elegans, and mice have prompted investigations into whether metformin has therapeutic utility as an anti-aging drug that may also extend lifespan. Indeed, clinical trials, including the MILES (Metformin In Longevity Study) and TAME (Targeting Aging with Metformin), have been designed to assess the potential benefits of metformin as an anti-aging drug. Preliminary analysis of results from MILES indicate that metformin may induce anti-aging transcriptional changes; however it remains controversial as to whether metformin is protective in those subjects free of disease. Furthermore, despite clinical use for over 60 years as an anti-diabetic drug, the cellular mechanisms by which metformin exerts either its actions remain unclear. In this review, we have critically evaluated the literature that has investigated the effects of metformin on aging, healthspan and lifespan in humans as well as other species. In preparing this review, particular attention has been placed on the strength and reproducibility of data and quality of the study protocols with respect to the pharmacokinetic and pharmacodynamic properties of metformin. We conclude that despite data in support of anti-aging benefits, the evidence that metformin increases lifespan remains controversial. However, *via* its ability to reduce early mortality associated with various diseases, including diabetes, cardiovascular disease, cognitive decline and cancer, metformin can improve healthspan thereby extending the period of life spent in good health. Based on the available evidence we conclude that the beneficial effects of metformin on aging and healthspan are primarily indirect *via* its effects on cellular metabolism and result from its anti-hyperglycemic action, enhancing insulin sensitivity, reduction of oxidative stress and protective effects on the endothelium and vascular function.

## Introduction

Metformin is a synthetic biguanide, orally effective and insulin sensitizing anti-diabetic drug, which for most patients is the first line anti-hyperglycemic for the treatment of type 2 diabetes mellitus (T2DM) (1). Metformin was first synthesized in 1922 and its development was based on knowledge from folk medicine that the active, but toxic, constituent from *Galega officinalis* (French lilac) that could treat ‘sweet urine’ was the guanidine, galegine (2, 3). Metformin was introduced to treat T2DM in France in 1958 and now after over 60 years is used on a daily basis by over 150 million people (4). For the majority of people metformin is not only an inexpensive drug as it is off patent but also a safe drug with the most prominent side effects being gastrointestinal-related that occur in about 20-30% of patients and can include abdominal pain, bloating, diarrhea, nausea and vomiting (5, 6). The GI-side effects are dose-dependent and do not usually limit its use; however, in approximately 5% of subjects these side effects can result in the need to switch to an alternative drug (5–9). The GI side effects can be linked to the accumulation of metformin in gut enterocytes and enhanced glucose utilization resulting in lactate generation, as well as metformin-induced changes in the microbiota (6, 10). More serious side effects include lactic acidosis, which is, however, very rare and is reduced by avoiding its use in patients with severe renal and liver dysfunction (9, 11–15). Several studies, but not all, have reported that the chronic use of metformin results in malabsorption issues and vitamin B12 deficiency, affecting between 6 and 30% of users. Thus, monitoring B12 levels will be essential if the use of metformin is expanded to become the panacea for all reasons and all diseases (16–20). On the positive side, metformin use leads to weight reduction in many patients, reduces HbA1c and its use is not associated with hypoglycemia. In addition, data from the Diabetes Prevention Program (DPP) indicates that metformin is effective in preventing the development of diabetes in patients with pre-diabetes and its use following a 10-year follow up associated with an average 2.1-kg weight loss (7). However, metformin has not been approved as a weight loss drug in part because its effects are highly variable and in women with PCOS despite weight loss visceral fat may not be reduced (21).

According to the WHO, diabetes and cardiovascular disease contribute approximately 22 million deaths annually (22). Given that metformin is so widely used, there is an opportunity to determine whether metformin does have anti-aging properties. This goal raises the question as to what really defines an anti-aging drug? We will use the definition that an *“anti-aging action refers to the prevention, and treatment of age-related diseases”* in other words *“positively affects healthspan, the period of life spent in good health and free of disabling diseases, or healthy lifespan”.* We acknowledge that the use of the term ‘healthspan’ is considered by some to be controversial as it cannot readily be quantified and therefore is subjective; however, the term is extensively used. Therefore, to be consistent, we retain its use, accepting the limitations that the term simply reflects a reduction in the risk of disabling disease. That reduction in itself could increase lifespan (23). Healthspan is thus distinct from extending life expectancy, or lifespan (longevity), which can be readily quantified, and is defined as *“the length of time an organism lives, or total lifespan”*.

The use of metformin in the setting of T2DM increased dramatically with the results from the United Kingdom Prospective Diabetes Study (UKPDS), a prospective, 20-year randomized, multicenter of patients with T2DM. The study, published in 1998, reported the cardiovascular (CV) benefits of the use of metformin for diabetes (24). It has long been assumed that the anti-diabetic benefits of metformin are due to its hepatic-mediated actions in human subjects and that other clinical benefits are secondary to its effects on glucose and lipid metabolism. We now know there are extra-hepatic sites of action of metformin with the gut being a major contributor to its clinical benefits as an anti-hyperglycemic drug *via* modulating glucagon-like peptide 1 (GLP-1) levels by a duodenal AMPK-mediated pathway, as well as effects resulting from modulating the microbiota (25–29). The benefits of metformin in reducing microvascular disease have been supported by a 10-year follow up of the original UKPDS report (30). Similarly, Johnson et al. accessed the Saskatchewan Health Administrative databases for a retrospective study and also reported reduced cardiovascular morbidity and mortality benefits of metformin monotherapy *versus* sulfonylurea monotherapy (31). Additional supportive pre-clinical and clinical evidence has been provided by Nesti and Natali (32). Collectively these data indicate that metformin can reduce both mortality and disease-accelerated aging of the cardiovascular system. Based on evidence from both clinical and pre-clinical studies metformin, independent of its anti-hyperglycemic actions, directly protects the endothelium (33–35). An accumulation of sometimes controversial data has opened the debate as to whether metformin’s favorable effects extend beyond its anti-hyperglycemic actions and include a role as an anti-aging drug that enhances healthspan and extends lifespan thus raising the hope that it could be the *“Fountain of Youth”* (26, 27, 29, 36–42).

In addition to its benefits as an anti-hyperglycemic agent, metformin also lowers the atherosclerotic burden in non-diabetic persons at risk of developing T2DM (1, 7). Interest in the potential clinical benefits of metformin has been extended to include repurposing for polycystic ovary syndrome (PCOS), preeclampsia, cancer, rheumatoid arthritis, malaria, and antibiotic and antiviral actions (43–48). Based on this ever-expanding catalogue of the benefits of metformin, the title of a review article refers to metformin as: *“Metformin, the aspirin of the 21^st^ century*—” (27).

In this review we summarize the published evidence that argues for, or against, an anti-aging effect of metformin. Furthermore we describe the putative anti-aging pathways that metformin activates and discuss with reference to ongoing clinical trials whether the use of metformin is translated into positive effects on both healthspan and lifespan. An important issue when evaluating the potential benefits of a drug in the treatment of any given medical condition is the strength and reproducibility of the evidence and a particular concern when translating data from pre-clinical studies of a drug to therapeutic efficacy (49–51).

## The Putative Relationship Between Metformin and Aging

Based on a systematic review of 53 studies, Campbell et al. concluded that independent of its therapeutic efficacy as an anti-diabetic drug, the use of metformin results in a reduction of all-cause mortality associated with diseases that accelerate aging, including cancer and cardiovascular disease (37).

The genes and cell signaling pathways related to the cell cycle, DNA repair cell death, mitochondria, immunity, nutrient signaling and the growth hormone Insulin Growth Factor-1 (IGF-1) mediated *via* the PI3K/AKT/mTOR (phosphoinositide 3-kinase/AKT (protein kinase B)/mammalian target for rapamycin) pathway have received extensive investigations as targets for anti-aging strategies (36, 52, 53). The potential for biguanides to be used as geroproetctors was described as early as 1980 (54). Metformin *via* its insulin sensitizing actions reduces insulin and thereby should also normalize IGF-1 levels (55, 56). The effects of metformin on IGF-1 are linked to metformin as an activator of AMP-activated kinase (AMPK) and inhibition of signaling through the mTOR pathway. mTOR is a highly conserved serine-threonine kinase that has important roles in the regulation of cell metabolism, including nutrient signaling and growth mediated by IGF-1 (57, 58). Signaling *via* mTOR is linked to accelerated aging and dysregulation of mTOR signaling is also linked to the progression of cancer, inflammatory and neurological diseases, as well as T2DM (59). AMPK is a key regulator of many cellular pathways that are linked to both healthspan and lifespan, including the benefits of calorie restriction. Thus, as an activator of AMP metformin has come under the microscope as a potential anti-aging drug and its potential role as an anti-aging drug promoted (36, 41, 60). Worthy of note is that the sensitivity to AMPK declines with age thus promoting the argument that activators of AMPK, such as metformin, could delay aging (61).

The lifespan and healthspan of invertebrates are extended by mutations in pathways such as the DAF-2, a gene that encodes for the IGF-1 pathway in the nematode Caenorhabditis elegans (C. elegans), or the mTOR signaling pathways that are linked to accelerating the aging process (60, 62–65). The addition of metformin to the diet can delay aging and increase life span in both C. *elegans* and rodents (66–68) (see **Table 1**). In C. *elegans* the beneficial effects of metformin on lifespan are, at least in part, argued to be mediated through changing the microbiome and modifying microbial folate and methionine metabolism and potentially due to an anti-oxidant action *via* transcription factors, SKN-1/Nrf2, a requirement for AMPK and the upstream serine-threonine kinase, liver kinase B1 (LKB1) (67, 71, 86). However, in these studies with C. elegans, very high concentrations of metformin, from 10 to 150 mM, were used (67, 86), and are the equivalent to a dose in humans of approximately 5 kg/day. Such doses in humans would be fatal and one would expect the same in C. elegans where toxicity is offset by the effects of metformin on the microbiome and inhibition of bacterial folate metabolism. Indeed, in the absence of bacteria, metformin shortened lifespan implying that it is the anti-bacterial action of metformin that explains the enhancement of lifespan in C. elegans (67). Furthermore, in old C. elegans all concentrations of metformin, 10, 25 and 50 mM, proved toxic and reduced lifespan (72). The enhanced toxicity to metformin in the older worms was linked to reduced mitochondria abundance and the reduced ability to generate ATP – see **Table 1** for details (72).

**Table 1:** Summaries of studies cited in the review with a focus on the effects of metformin on lifespan.

Study	Protocol	Results
Bannister et al. (69)	• T2DM patients treated with metformin or sulfonylurea monotherapy were compared to age- and sex-matched non-diabetic control groups in a retrospective observational analysis from the UK Clinical Practice Research Datalink.	• Patients prescribed sulfonylureas had lower survival rates than non-diabetic controls and diabetic patients prescribed metformin. • Diabetic patients taking metformin had more co-morbidities, but their survival rates were comparable to the non-diabetic control group. • Conclusion: Metformin extends healthspan, but not lifespan in humans.
Willcox and Willcox (70)	• Okinawans have long lifespans. Epidemiological data on older Okinawans, on a caloric restriction-like diet for approximately half their lives, (caloric restriction (10-15%), consumption of foods that mimic biological effects of calorie restriction, and phenotypic evidence consistent with caloric restriction (low body weight, and BMI).	• Caloric restriction likely contributed to the extended healthspan and lifespan of the Okinawans.
Onken and Driscoll (71)	• The effects of metformin on the healthspan and lifespan of the nematode Caenorhabdatis elegans and linked to activation of the serine-threonine kinase LKB1 and AMPK. Benefits of metformin were dependent on expression of the stress-responsive SKN-1/Nrf2, but independent of the insulin-signaling pathway?	• Exposure to 50 mM, but not 1 or 10 mM, metformin significantly enhanced survival of C. elegans by 27%, significantly right-shifted the survival curve and promoted ‘youthful’ mobility. The effects were not observed in the EAT-2 DR model of calorie restriction or in models with deficient AMPK, LKB1 or SKN-1/Nrf2. • Conclusion: Metformin via AMPK/LB1/SKN-1/NrF2 axis extends lifespan in C. elegans.
Espada et al. (72)	• The effects of metformin on lifespan were studied in different age groups of young C.elegans: AD1 -young adults AD4 –adults declining in reproductive potential AD8 –Middle aged AD10 -Old	• Exposure to 10, 25 and 50 mM metformin in AD1 and AD4, 25 and 50 mM decreased life expectancy in AD8, and in old C.elegans (AD10) all concentrations proved toxic. Toxicity was linked to a decrease in mitochondria and lower levels of ATP in the older worms that resulted in enhanced toxicity to metformin. Mutants that were resistant to metformin toxicity had higher mitochondria content and expression of complex 1.
Anisimov et al. (66); Also see Anisimov et al. (73) and Anisimov et al. (74)	• Anisimov et al. (66): Studies in old *vs.* young mice • 160 female Swiss-H Rappolovo (SHR) mice were in [100 mg/kg] daily, *vs.* tap water without metformin [control]. • Anisimov et al. (74): Comparison of treatment with metformin: • a) started at 3 months of age. • b) started at 9 months of age. • c) started at 15 months of age.	• When added to the diet of SHR mice, metformin slowed aging and increased lifespan, but did not lower incidence of spontaneous tumors. The anti-aging effectiveness of metformin was reduced in older mice. • Conclusion: Metformin effects on lifespan in mice are age-dependent. • Anisimov et al. (74): Metformin extended lifespan by 14% when started at 1 month but at 9 months only by an insignificant 6%, and at 15 months was ineffective. • In the 2003 paper Anisimov et al demonstrate a comparable lifespan extending effects in mice and rats with the biguanides, phenformin and busoformin that also attenuated tumor development suggesting to the authors that they are potential geroprotetors.
Alfaras et al. (75)	• Intermittent [either every other week (EOW) or two weeks out of 4 (2WM)] treatment of aged male C57/BL6 mice for 17 weeks with 1% metformin in diet	• Intermittent metformin treatment did not lead to early mortality. • EOW metformin resulted in weight loss and improved insulin sensitivity, but not lifespan extension. • Compared to controls evidence of increase in renal lesions in EOW and 2WM mice.
Martin-Montalvo et al. (76)	• Cohorts of middle-aged mice were fed either a normal diet or a standard diet supplemented with 0.1% (w/w) or 1% (w/w) metformin, for the remainder of their lives.	• In male mice, long-term treatment with 0.1% metformin w/w resulted in serum levels of 450 μM increased lifespan by 4.15%, and reduced NF-κB in the liver. • A higher dose of 1% metformin w/w resulted in serum levels of 5 mM and was toxic and resulted in a 14.4% reduction in the average lifespan of mice. • Parallel studies in mouse embryonic fibroblasts demonstrated activation of AMPK without affecting mitochondria electron transport activity. • Conclusion: Metformin extends lifespan in mice, but dose-dependent.. • Note: Comments on the western blot data are available on pubpeer.
Strong et al. (77)	• The National Institute on Aging Interventions Testing Program (ITP) dataset evaluated 0.1% metformin and rapamycin (14 ppm) effects on lifespan in mice.	• Metformin alone did not increase lifespan, but in combination with rapamycin, a benefit was reported. The authors speculate that the ‘benefit’ of metformin is via offsetting the negative effects of rapamycin on metabolism. • Conclusion: Metformin does not extend lifespan in mice.
Smith et al. (78)	• Male Fischer rats from 6 months of age were subjected to either a calorie restricted (CR) diet (70%), or dietary metformin (300 mg/kg/day) *versus* a control group. Metabolic parameters, body weight and lifespan, were determined.	• Based on Kaplan-Meier survival plot analysis, metformin did not extend lifespan *versus* control, whereas CR delayed early mortality. • Conclusion: Metformin is not a calorie-restriction mimetic (CRM)
Kulkarni et al. (79)	• MILES Trial commenced October 2014. A crossover, double-blinded, study with 14 elderly subjects with impaired glucose control and each serving as their own control. Subjects were treated with 1700 mg/day metformin for 6 weeks and transcriptomic studies of biopsies from skeletal muscle and subcutaneous adipose tissue were conducted.	• 647 genes were differentially expressed in muscle *versus* 146 in adipose tissue affecting both metabolic and non-metabolic pathways • Changes in DNA repair and, since repair function declines with age, data suggests an anti-aging action. Similar results for changes in collagen gene expression. • In adipose tissue changes were observed in fatty acid and lipid metabolism as reflected by PPAR and SREBP signaling. • In skeletal muscle changes in pyruvate metabolism, NAD biosynthesis, and down regulation of PARP1 were observed. The latter suggesting an effect on mitochondria function. • Conclusion: Preliminary evidence implying metformin effects on transcription and pathways affecting healthspan and lifespan.
ai. Gerstein et al. (80)	• Analysis of 237 biomarkers from 8401 participants with diabetes or impaired glucose tolerance in the ORIGIN trial (Outcome Reduction with Initial Glargine Intervention).	• ai. Analysis identified 10 biomarkers that identified dysglycemic subjects at higher *versus* lower CV risk.
aii. Gerstein et al. (81)	• Analysis of the biomarker profile of the 28% of the 8,401 participants in the ORIGIN trial who were receiving metformin.	• aii. Subjects taking metformin also had higher GDF15 (Growth Differentiation Factor 15) levels and lower CV outcomes.
b. Tanaka et al. (82)	• b. Proteomic analysis of plasma from 240 healthy, disease-free, subjects in the age range of 23-93 years.	• b. Plasma levels of GDF15 correlated with chronological age.
c. Coll et al. (83)	• Metformin *vs.* placebo treatment of fat fed mice either expressing GDF15 or lacking the receptor, GRAL, or treated with a GRAL antagonist.	• Weight loss effect shown to be dependent on expression of the GDF15 receptor, GRAL, (glial cell-derived neurotrophic factor family receptor alpha-like) whereas the antihyperglycemic effect of metformin was independent of the GDF15-GRAL pathway.
d. Modi et al. (84); Wischhusen et al. (85)	• d. Review of literature re. expression levels of GDF15 in various cancers and signaling pathways via EGFR and PI3K, Akt pathways.	• GDF15 a putative prognostic indicator of tumor progression and therapeutic target and raising the question as to whether GDF15 serves as a tumor suppressor, or as a promoter and is a target for the treatment of cancer.

The same pathways studied in C. elegans have also been investigated in mammals, where lifespan and healthspan were prolonged through modifying the ‘aging’ pathways or through drugs, including metformin (60, 87). As for C. elegans, metformin use in humans will change the microbiome. This action is an important determinant for the anti-hyperglycemic therapeutic effects, the gastrointestinal side effects and possibly the anti-aging effects of metformin (88, 89). Metformin has a bioavailability of approximately 50% with the unabsorbed drug exiting in the stool and following a dose of up to 2550-mg/day; the concentration of metformin in the gut could reach mM levels. In addition, and also *via* gut-mediated action, metformin enhances the release of glucagon-like peptide-1 (GLP-1). It is the release of GLP-1 that contributes substantially to the antihyperglycemic effects of metformin (26, 90, 91). Additional support for the importance of gut-mediated mechanisms for metformin’s actions is the knowledge that IV administered metformin is ineffective in lowering hyperglycemia and was demonstrated using a hyperglycemic clamp technique to determine the relationship between plasma metformin concentration and hepatic glucose production and disposal in T2DM patients (92).

The recognition of the role of both the gut microbiome and the release of GLP-1 as important contributors to the anti-diabetic effects of metformin has been a significant advance in understanding how metformin mediates its therapeutic actions including the contribution to the variable weight loss that is associated with metformin (93). An additional factor in weight loss is the novel cytokine, Growth Differentiation Factor 15 (GDF15), which is a member of the transforming growth factor β superfamily and also known as macrophage inhibitory cytokine-1 (MIC-1. Secretion of GDF15 has been linked to a number of physiological and pathophysiological functions and expression levels are elevated in response to stress stimuli and associated with stress pathway transcription factors, CHOP (C/EBP homologous Protein) and ATF4 (activating transcription factor 4) (94–96). As a result of an analysis of data from the ORIGIN trial (Outcome Reduction with Initial Glargine Intervention), GDF15 was identified as a biomarker linked to positive CV outcomes and also elevated in those patients receiving metformin (80, 81). The association between metformin and GDF15 was strengthened by data showing that metformin prevented weight gain in high fat fed mice, but not in mice lacking GDF15, or lacking its receptor, glial cell-derived neurotrophic factor family receptor alpha-like (GFRAL), or following treatment with a GRAL antagonist; however, GDF15 was not required for the antihyperglycemic effects of metformin (83). A number of studies have reported a positive correlation between the use of metformin and enhanced levels of GDF15 (97). Of additional interest and based on a proteomic analysis of the plasma from 240 healthy, disease-free, humans in the age range 22-93 years-old, GDF15 was identified as the protein most positively correlated with chronological age and also known to reduce appetite *via* an action in the hind-brain (82, 95). Collectively, these data suggest a link between the putative anti-aging effects of metformin, weight loss and CV benefits that are distinct from the effects of metformin on glucose homeostasis. GDF15 also signals through the epidermal growth factor (EGF) family of receptors and the PI3K, and AKT signaling pathways raising the question whether an elevation of GDF15 is necessarily protective (94, 98). Elevated levels of GDF15 have been linked to tumor growth and poor prognosis in cancer patients raising the question as to whether GDF15 serves as a tumor suppressor or promoter and a target for the treatment of cancer (84, 85, 99). See **Table 1**.

## AMP-Activated Protein Kinase

**Figure 1:**
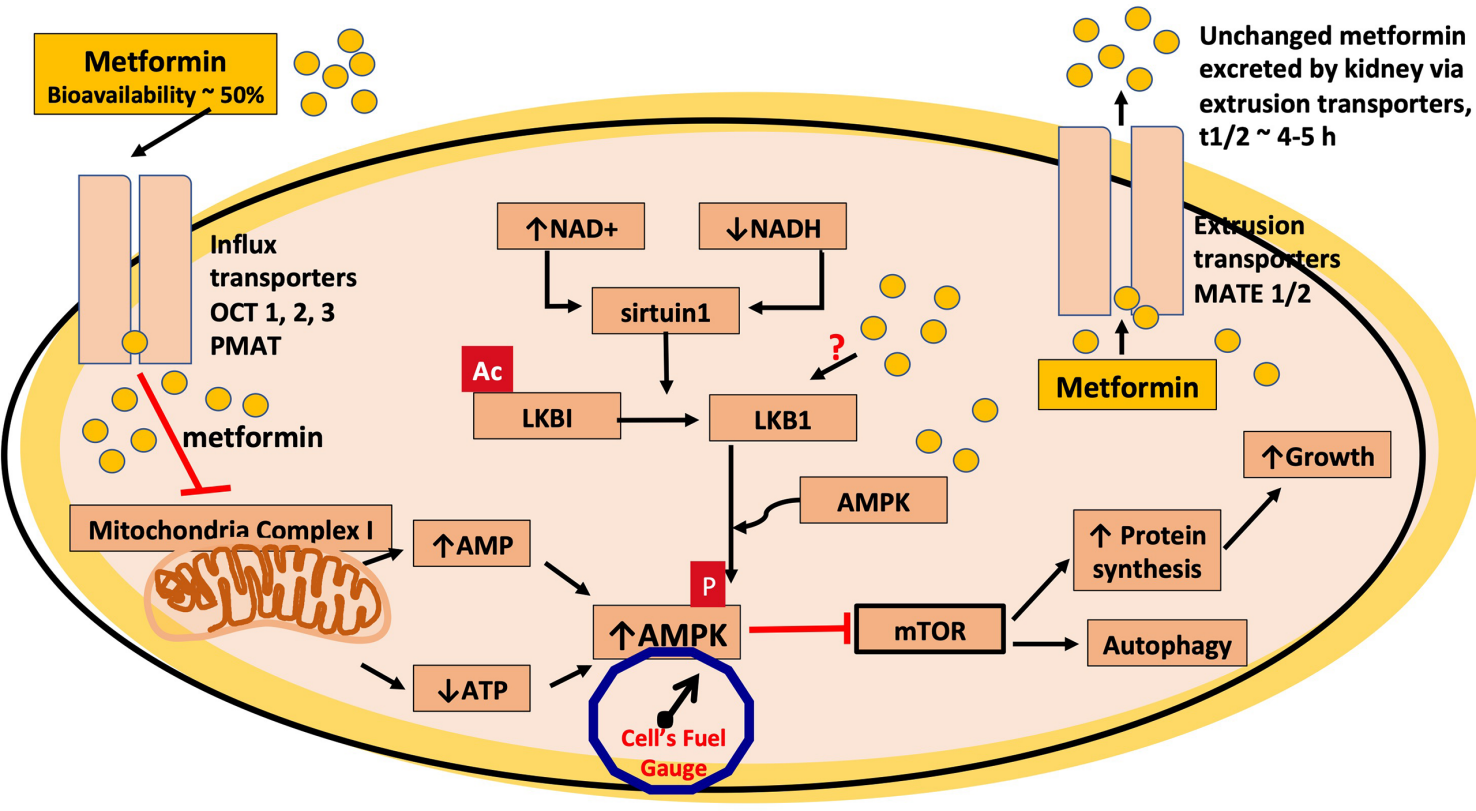
Metformin inhibits mitochondrial complex 1. In this schematic, metformin is transported into the cell via the organic cation transporters, OCT 1, 2 and 3 and the plasma membrane monoamine transporter (PMAT). The transport of metformin out of the cell is mediated through the multidrug and toxin extrusion (MATE1/2) transporters. Metformin is thought to mediate most of its cellular effects via activation of AMPK and, in this schematic, metformin inhibits the electron transport chain of mitochondrial complex 1, which leads to a reduction in ATP levels, increasing the AMP/ATP ratio, thus increasing AMPK activation and also reduces the generation of reactive oxygen species (ROS). AMPK activation leads to an inhibition of the mTOR pathway, which would contribute to the antitumor effects of metformin. Metformin also has been shown to activate AMPK via the serine-threonine liver kinase B1 (LKB1) where phosphorylation (p) (activation) of AMPK occurs. The protein product of SIRT1, sirtuin1, is an upstream deacetylase, which activates LKB1 via deacetylation as indicated in the figure by loss of ac, at times of cellular stress and decreased cellular energy, when NAD+/NADH ratio is high and is also a putative site of action for metformin.

AMPK functions as an energy sensor that coordinates multiple protective and energy-conserving signaling pathways including the pathways activated by caloric restriction and referred to as the fuel gauge of the cell by serving as an energy sensor (100, 101) (**Figure 1**). AMPK is activated through metabolic stress and acts as a cellular regulator of lipid and glucose metabolism (102, 103). Hepatic gluconeogenesis is inhibited by AMPK activation, which also enhances insulin sensitivity, muscle glucose absorption, and fatty acid oxidation (103, 104). Additionally, metformin inhibits the inflammatory response through nuclear factor κB (NFκB) inhibition *via* pathways involving AMPK (104, 105) (**Table 2** and **Figure 1**). An increase in the activity of AMPK would also explain the protective effects of metformin on endothelial function *via* the activation of endothelial nitric oxide synthase (eNOS) thereby countering the negative effects of a diabetic milieu on cardiovascular function (119). AMPK inhibits signaling *via* mTOR and through this action could contribute to the reduced incidence of some cancers that has been associated with the use of metformin: see **Table 2**. Furthermore, reduced sensitivity to AMPK activation with age or disease could result in reduced healthspan and lifespan (61). Although these data support the argument that metformin enhances both healthspan and lifespan, caution is needed because of the high concentrations of metformin, well above therapeutic levels in humans that have been used in some of the pre-clinical *in vitro* studies.

**Table 2:** Summaries of studies that focus on cellular actions of metformin to activate AMPK, reduce generation of reactive oxygen species, improve vascular function, mediate anti-inflammatory effects and the potential to treat cancer and neurodegenerative diseases.

Study	Protocol	Results
Mather et al. (106)	• A 12-week study of metformin-naïve patients with T2DM receiving 500 mg metformin bid, or placebo, with endothelial function assessed via use of forearm strain-gauge plethysmography following the intra-brachial artery administration of the endothelium-dependent vasodilator (EDV) acetylcholine *versus* responses to the endothelium-independent vasodilators (EIDV), sodium nitroprusside and verapamil.	• Metformin improved EDV but not EIDV. Results interpreted as reflecting metformin-mediated reduction in insulin resistance (as determined by measuring whole-body insulin resistance, HOMA-IR) improves endothelial function and offsets diabetes-associated vascular disease.
Ouslimani et al. (107)	• The effects of 10 μM metformin on ROS production in bovine aortic endothelial cells (BAECs) that were either non-stimulated or stimulated by high glucose levels, or by angiotensin II (ATII).	• Metformin substantially reduced intracellular ROS levels in both non-stimulated and glucose- or ATII-stimulated cells. • NADPH oxidase and mitochondrial respiratory chain-induced activities were also decreased by metformin.
Ding et al. (108), Aljofan and Ding 2010 (109)	• Studies of the effects of high glucose on expression of dimeric and monomeric, eNOS, oxidative stress, NADPH oxidase and cyclooxygenase-2 (COX-2) in murine microvascular endothelial cells (MMECs).	• High glucose uncouples eNOS resulting in an increase in the ratio of monomeric to dimeric eNOS and thereby enhances oxidative stress together with enhanced expression of NADPH oxidase (p22phox) and COX-2 with resultant reduced generation of nitric oxide.
a. Arunachalam et al. (110)	• a. Potential protective effects of metformin (50 μM) on high-glucose induced oxidative stress in MMECs and the role of the decacetylase sirtuin1 on endothelial cell senescence.	• Expression of sirtuin-1 required for metformin protection of endothelial cells against high glucose-induced senescence.
b. Ghosh et al. (111)	• b. Effects of 50 μM metformin on endothelium-dependent vasorelaxation (EDV) in aortae from mouse model, db/db (leptin receptor mutant) of T2DM and obesity. • Effects of metformin on eNOS phosphorylation in MMECs exposed to normal *versus* high glucose cell culture conditions.	• Metformin improved EDV in vascular tissue from diabetic mice. • A 3 hour exposure to 50 μM metformin reduced high-glucose induced reduction in phosphorylate in eNOS and Akt. Data also suggest effects of metformin are partially dependent on AMPK activation. • Collectively the data from studies of metformin and endothelial function indicate: a. Metformin protects endothelial function in patients with diabetes and • b. pre-clinical data supports a direct protective effect on the endothelium via reducing oxidative stress and hyperglycemia-induced endothelial cell senescence.
Stephenne et al. (112)	• Rat, mouse and human hepatocytes in culture were exposed to 500 μM to 5 mM metformin and AMPKα activity, and mitochondrial oxygen consumption rates were measured at different time points.	• Metformin increased AMPK activity in rat and human hepatocytes and was associated with an increase cellular AMP:ATP ratio. • Hepatic AMPK activation by metformin resulted from lower cellular energy due to AMPK-independent inhibition of the mitochondrial respiratory-chain complex by metformin. • Conclusion: AMPKα1 was activated in human hepatocytes, whereas both AMPKα isoforms were activated in rat hepatocytes stressing the importance of species differences.
Hattori et al. (105)	• The effects of metformin on NF-κB activation and expression of NF-κB -mediated genes were studied in human vascular endothelial cells (HUVECs) in culture.	• Dose- and time-dependent activation of AMPK from 1 to 10 mM metformin and inhibition of proinflammatory and adhesion molecule genes induced by cytokines through suppression of NF-kB activity. • Conclusion: Metformin has anti-inflammatory effects but requires high concentrations.
Evans et al. (113)	• Case-control study tested hypothesis that patients using metformin have a reduced risk of cancer. Study utilized diabetes clinical information system (DARTS) and prescription drug database (MEMO) for Tayside, Scotland. 314,127 patient files were reviewed for the period, 1993-2001.	• Analysis indicates a reduced risk of cancer and a suggestion of a dose-dependent effect. • Speculate a link between the putative anti-cancer effect of metformin and its action to activate AMPK and role of the upstream serine-threonine kinase, LKB1, a known tumor-suppressor. • Conclusion: Epidemiological evidence that metformin reduces incidence of cancer.
Nair et al. (114)	• The effects of mM concentrations (5 to 20) of metformin were given to Panc 1 human pancreatic cancer cells in culture.	• mM metformin inhibits mTOR signaling as reflected by a reduction in phosphorylated Akt and mTOR. • Metformin inhibits activation of mTOR through down-regulation of the Sp1, Sp3 and Sp4 transcription factors transcription and IGF-1R. • Conclusion: Metformin targets genes and signaling pathways important for tumor migration, invasion, proliferation and survival.
Wang et al. (115)	• Microvessel density, vascular maturity and function, lung metastasis, and chemosensitivity were compared in metformin-treated *vs.* untreated mice using constructed metastatic breast cancer models.	• Metformin decreased microvessel density, leakage, and hypoxia while increasing vascular mural cell perfusion as compared to a control group. • Conclusion: Vascular effects mediate beneficial effects of metformin on breast cancer.
Ng et al. (116)	• In the population-based Singapore Longitudinal Aging Study, 365 diabetic subjects were monitored for 4 years in a cross-sectional and longitudinal multivariate analysis, evaluating odds ratios of association of metformin use with cognitive decline (Mini-Mental State Exam ≤ 23).	• Metformin use for more than 6 years was linked to a decreased risk of cognitive impairment. • Conclusion: There is a potential benefit of metformin for delaying cognitive decline and thereby extending healthspan.
Fatt et al. (117)	• Effects of metformin on proliferation, self-renewal and differentiation of adult neural stem cells (neuronal progenitor cells, NPC) from mice. • Primary neurospheres were studied in the presence or absence of 1 μM metformin. • Adult mice injected (i.p) with 200 mg/kg metformin for 7 days. Sections of supraventricular zone taken and total number of BrdU cells quantified. • *In vivo* studies to determine role for the stress-response gene.in TAp73-/- mice.	• 1 μM metformin enhanced the self-renewal and differentiation of NPCs. • Neuronal differentiation was dependent on AMPK/atypical PKC and cyclic AMP response element-binding protein (CREB or CBP). • Metformin mediated self-renewal and proliferation of NPCs via activation of Tap73 gene. The protein p73 is related to the tumor suppressor, p53. • Conclusion: Metformin enhances neurogenesis suggesting a role for metformin in neurodegenerative diseases.
Ma et al. (118)	• *In vivo* study to investigate effects of chronic treatment of metformin (250 mg/kg/day by gavage, 12 weeks) on hippocampal neurogenesis and learning and memory of high fat diet obese mice. • Memory and learning assessed based on exploration time. • Fecal pellet study to determine contribution of microbiota. • Numbers of hippocampal neurons determined in newborn pups.	• Obesity resulted in a decline in memory and learning in adult mice that was prevented by metformin. • Metformin enhanced neurogenesis in hippocampus. • Expression of inflammatory cells was higher in fat-fed mice and decreased by metformin. • Beneficial effects of metformin mimicked by fecal pellet transplant from metformin-treated to non-treated mouse. • Conclusion: Important role for Gut-Brain axis in mediating effects of metformin on neurogenesis.

AMPK is regulated by two upstream serine threonine AMPK kinases: LKB1, and Ca2+/calmodulin-dependent protein kinase kinase β (CaMKKβ) and is also positively regulated by the nicotinamide adenine dinucleotide (NAD+) deacetylase, sirtuin-1. Sirtuin-1 is the protein product of the putative anti-aging gene SIRT1, which targets lysine residues in proteins, including histones and the tumor-suppressors, LKB1 and p53 (120, 121). In endothelial cells, metformin has been shown to enhance phosphorylation and increase the activity of LKB1 (122). Senescence is a major contributor to aging and the development of cardiovascular disease (123), and sirtuin-1 expression is required for metformin to protect endothelial cells against hyperglycemia-induced senescence (110). Furthermore, *in silico* analysis suggests that metformin could directly activate SIRT1 (124).

Of significance and supporting a potential anti-cancer action of metformin is that LKB1 is a tumor suppressor and that mutations in LKB1 are observed in numerous cancers that result in the reduction of the inhibitory effect of the LKB1/AMPK pathway on the pro-proliferative signaling *via* mTOR (125, 126). Collectively these data provide a link between metformin, AMPK, LKB1, sirtuin-1, and cellular mechanisms that could enhance both healthspan and lifespan by reducing both cellular senescence and the activation of pro-proliferative pathways.

## AMPK as a Target for Metformin

Considerable attention has been placed on the liver and skeletal muscle as the primary sites of action of metformin resulting from the activation of AMPK secondary to an inhibitory action on mitochondrial complex 1. This inhibition would in turn decrease the ATP/AMP ratio activating AMPK (127–129). A small increase in the level of AMP enhances AMPK *via* three mechanisms: (i) allosteric activation by AMP binding to the Υ subunit (ii) promotion of phosphorylation of Thr-172, and (iii) inhibition of dephosphorylation of Thr-172 (130). Furthermore, an increase in AMP also decreases adenylate cyclase activation and thereby lowers glucagon release (129). This mechanism is an attractive hypothesis to explain how metformin mediates a multitude of cellular effects *via* AMPK (100). Evidence that metformin targets mitochondrial complex 1 is supported by data from several studies (112). The role of complex 1 inhibition in mediating the effects of metformin is summarized in **Figure 1**, and much of the published literature assumes this inhibition is indeed the mechanism whereby the drug mediates its effects *via* AMPK, and the reduction of the generation of ROS from complex 1 (131). However, several other mechanisms to explain the cellular actions of metformin have also been advanced [see (39, 132)].

As pointed out by Fontaine, a problem associated with accepting the argument that the therapeutic effects of metformin result from inhibition of complex 1, as depicted in **Figure 1**, are the high concentrations used in the cell culture studies described by El Mir et al. and Owen et al. (127, 128, 133). For instance, activation of AMPK by metformin has been observed at much lower metformin concentrations in comparable cell culture protocols using metformin concentrations that are within the upper limits of its therapeutic plasma levels (4, 134). In addition, studies have also reported AMPK/LKB1 independent effects of metformin on gluconeogenesis (135). On the other hand and based on thermodynamic considerations, Owen et al. (128) argue that with a mitochondrial membrane potential of – 180mV metformin could accumulate 1000 fold thus potentially providing a concentration within the mitochondria that would inhibit complex 1 [see also Vial et al. (136)]. Partial support for this hypothesis has been provided by Chien et al. who used a cell culture protocol with human embryonic kidney (HEK) cells that were overexpressed with the organic cation transporter 1 (OCT1) and exposed for 60 minutes to 5 μM ^14^C-metformin (137). Chien et al. found that higher concentrations of metformin were found in the HEK cells with overexpressed OCT1 *versus* control HEK cells (268+/-11 *vs.* 26.4+/-11 μM) (137). On the basis of this enhanced uptake, Chien et al. argue that metformin can be trapped in intracellular organelles such as microsomes and mitochondria (137). In a similar protocol, the *in vitro* and *in vivo* effects of metformin on mitochondria function were investigated *in vitro* in primary murine hepatocytes and Hepa 1-6 cells (murine hepatoma cells derived from the BW7756 tumor) and an *in vivo* protocol with high-fat diet (HFD) treated mice (138). In the *in vitro* studies, Hepa1-6 cells were exposed to either 75 or ‘supra-pharmacological’ 1000 μM metformin; however, even following exposure to 1000 μM, no inhibitory effects were seen on the activities of complex I-IV, and the subcellular distribution of metformin indicated that metformin remained primarily in the cytosol with levels in mitochondria less than 70 μM (139). The *in vivo* arm of the study involved treatment of the HFD mice, including AMPKα1/2 knockout mice, with 50-mg/kg/day metformin in the drinking water and then assaying hepatocytes *in vitro* with the results indicating that complex 1 activity, mitochondria density and mitochondrial DNA were all increased *via* an AMPK-dependent mechanism (138). It is also worthy of note that the IC50 for metformin to inhibit mitochondrial complex 1 is 19–66 mM (140, 141).

Collectively these data cast doubt as to the contribution of inhibition of complex 1 as the primary target and explanation for the therapeutic effects of metformin both as an anti-diabetic and as an anti-aging drug. This conclusion is also supported in reviews (133, 142), and by clinical data from T2DM patients treated with metformin that show normal mitochondrial complex 1 respiration in skeletal muscle biopsies (143). As discussed below, it is important to consider the pharmacokinetic properties of metformin when extrapolating data from *in vitro* studies to potential benefits when used clinically.

## Metformin and Mitochondrial Function

Mitochondria play a critical role in oxidative metabolism and a link has been made in patients between obesity, insulin resistance, and defective mitochondria oxidative processes that results in a buildup of toxic intermediate metabolites (144). Several studies have also provided evidence that mitochondrial function is impaired in diabetes and thus the additional insult of metformin-induced dysfunction would be detrimental to patients with T2DM (145, 146), as was seen with the effects of metformin on old (AD10) C. elegans as previously discussed (72). Complex 1 activity in mitochondria from skeletal muscle biopsies was lower in T2DM and obese subjects compared to diabetic lean subjects (147). Although these data do not entirely negate the argument that metformin mediates its anti-hyperglycemic actions *via* an inhibitory action on mitochondria complex 1, the evidence strongly suggests that further inhibition of function would worsen, and contribute to, the toxic effects of metformin, such as lactic acidosis, that are, albeit very rare (11). As suggested by Wang et al., metformin may play an important role in the *‘quality-control’* regulation of mitochondria and shift the cellular dynamics to a healthy population *via* mitophagy and eliminate damaged mitochondria (138) [see also (148)]. AMPK has also been implicated in the regulation of mitochondrial biogenesis, thus providing another link between metformin, AMPK, and improved mitochondrial function (149, 150). Mitochondrial function declines with age due either to ROS and/or an accumulation of mutations in mitochondrial DNA (151). If metformin can offset the decline in mitochondrial function, as some studies suggest, then this effect could contribute to enhancing both healthspan and lifespan (138, 149, 150). Interestingly, both metformin and the anti-oxidant, resveratrol, inhibit ROS-associated mitochondria fission (152). Numerous studies have also reported that resveratrol has anti-aging effects in several species with its effects, as also described for metformin, linked to the deacetylase, sirtuin-1 (153). That said, the concentrations of resveratrol present in the diet, e.g. in wines, are far below the amounts that might have any effect on aging. Collectively, these data suggest an important role for metformin in the regulation of mitochondria function that could link to beneficial effects on both healthspan and lifespan.

## Pharmacokinetic Properties of Metformin

In order to extrapolate data from *in vitro* studies to *in vivo* therapeutic effectiveness, it is important to review the pharmacokinetic properties of metformin (34, 133, 154–156). When prescribed in the usual oral dosage range of 250 to ~2550 mg/day for the treatment of T2DM and in the absence of reduced renal function, it is unlikely that peak plasma levels of metformin would exceed 15-20 μM, with trough levels of between 1-5 μM, and a plasma half-life of approximately 3 to 5 hours. Metformin is a strongly basic hydrophilic drug with a pKa of approximately 11 is not metabolized and is excreted principally by the kidney. The expression levels of the saturable organic cation transporters will determine the rate of absorption of metformin from the gut and the subsequent cellular distribution and excretion of metformin. There are three distinct classes of organic cation-selective transporters that can be utilized by metformin: Organic Cation Transporter (OCT) 1, 2 and 3 (SLC22A1, A2, A3); Plasma membrane Monoamine Transporter (PMAT; SLC29A4); and Multidrug And Toxin Extrusion protein (MATE) 1 and 2 (SLC47A1, A2), to enter and leave cells (**Figure 1**) (157). A high expression levels of influx transporters in cells might allow a much higher accumulation of metformin resulting in selective toxicity and cell death as has been postulated to explain the anti-cancer effects of the drug (158). However, with a plasma half-life of approximately 3 to 5 hours, and subject to the schedule of dosing, it is unlikely, that other than in gut enterocytes, a significant cellular accumulation of metformin occurs when the drug is used for the treatment of T2DM (34, 155, 156, 159, 160).

It is important that results from *in vitro* studies using high micromolar and millimolar concentrations of metformin, often for long-exposure times, are looked at critically as such data may not be readily translated to effects when used in humans. A useful comparison of the doses of metformin used therapeutically *versus* pre-clinical studies was made by Badrick and Renehan, indicating that for pre-clinical studies done *in vivo*, the doses ranged from x2 to x45 therapeutic doses and that for studies done *in vitro*, concentrations ranged from x25 to x1000 higher than those observed in patients (161).

Justification for the use of high concentrations of metformin in *in vitro* studies has been provided. Thus, Onken and Driscoll (71) reported that the anti-aging actions of metformin in C. elegans were only seen with 50 mM metformin and not lower concentrations and stated: *“C. elegans has a highly protective cuticle and intestinal lining that generally limit drug uptake such that it is not unusual for polar drugs to be applied at a concentration 1000 fold higher than their predicted affinity for the target; physiological levels of drug in animals are anticipated to be much lower.”* [(71); see **Table 1**]. Although this rationale may be true for *C. elegans*, the absorption and distribution of metformin in mammals is also dependent on organic cation transporters (OCTs) and therefore a similar argument should be applied to other species unless it can be shown that C. elegans has a very low expression level of influx transporters and high expression of extrusion transporters thus justifying the use of high mM doses. Similarly, others have defended drug levels as matching plasma levels in humans and not acknowledged the short half-life of metformin; a drug that is not metabolized, for which plasma levels rapidly drop as metformin is extruded from cellular sites and excreted by the kidney.

## Metformin and Mortality in Diabetic Patients

Data from multiple studies indicate that patients with T2DM who are prescribed metformin have improved survival rates when compared to non-diabetic controls. In a study using retrospective observational data from the UK Clinical Practice Research Datalink, T2DM patients on metformin or sulfonylurea monotherapy were compared to non-diabetic control groups that were age and sex matched (69) (see **Table 1**). The data show that matched non-diabetics had 15% lower median survival times *versus* diabetics on metformin monotherapy and that those diabetics on sulfonylurea monotherapy had a 38% lower survival time compared to the metformin group (37, 69). Collectively, these data imply a clear mortality benefit associated with the use of metformin. However, the lower survival rate of those in the sulfonylurea-treated group might reflect a negative effect of sulfonylurea drugs on mortality as has been reported in subsequent studies (162). On the other hand, a recent systematic review concluded that metformin significantly lowered all-cause mortality and cardiovascular events in patients with T2DM and mild/moderate chronic kidney disease (163). Similarly, a meta-analysis with the objective of determining the cardiovascular benefits of metformin in combination with newer anti-diabetic drugs including incretins (GLP-1 agonists), dipeptidyl peptidase-4 (DPP-4) inhibitors, and sodium-glucose co-transporter 2 (SGLT2) inhibitors revealed both positive and neutral effects of adding metformin (164, 165). Nonetheless, in the absence of appropriately designed placebo-driven randomized control studies (RCTs) that include comparison with GLP-1 agonists, DPP-4 and SGLT2 inhibitors, doubt persists if, in fact, metformin does reduce the cardiovascular risk associated with diabetes (166).

## Metformin and Endothelial Function and Cardiovascular Disease

The endothelium plays a critical role in the regulation of cardiovascular function and not least as a source of the important signaling molecule, nitric oxide (NO). Furchgott and Zawadski were the first to report that an intact and undamaged endothelium was essential in order for acetylcholine to mediate a vasodilator response (167). We now refer to acetylcholine as an endothelium-dependent vasodilator, and we also recognize that endothelial dysfunction, which can be defined as a reduced vasodilator response to acetylcholine, is an early prognostic indicator for the onset of cardiovascular disease (168–171). Robert Furchgott, together with Louis Ignarro and Ferid Murad, shared the 1998 Nobel Prize for Physiology and Medicine *“for their discoveries concerning nitric oxide as a signalling molecule in the cardiovascular system*”. Remarkably it was the much earlier microscopy work of Dr. Rudolf Altschul in Saskatoon Canada that recognized the link between hypercholesterolemia and the pathological changes in the endothelium. Based on his comparisons of blood vessels of patients who died from cardiovascular disease *versus* other causes Dr. Altschul stated in the preface to his 1954 book:

*“Because the great majority of heart diseases is caused by coronary thrombosis, which is a lesion of the vessels supplying the heart proper, we may as well cancel the first word in “cardiovascular” and conclude that, in North America most people who die a natural death succumb to a vascular disease. Blood vessels, however, are primarily endothelial tubes with secondary, accessorial walls, and, therefore, it may be postulated that the endothelium has a great importance in our life and that its failure will cause the death of many of us.*

*It has been said that one is as old as one’s arteries. In view of the supreme importance of the endothelium in arterial function I should like to modify, or rather simplify this statement by saying that one is as old as one’s endothelium”* (172).

The obvious question is*: “Does metformin exert a direct anti-aging effect on the endothelium?”*. Based on critical reviews of the literature, the answer appears to be *“yes*”, although the clinical data could be interpreted as secondary to metformin’s actions to enhance insulin sensitivity and lower blood glucose levels (34, 106, 173). There is an extensive literature based on data from pre-clinical and clinical studies that indicates metformin protects endothelial function from the effects of diabetes (33). Positive clinical data have been provided by Mather et al. who used forearm strain-gauge plethysmography to assess the effects of metformin on forearm blood flow following the intra-brachial artery administration of acetylcholine *versus* responses to the endothelium-independent vasodilators, sodium nitroprusside and verapamil, in patients with T2DM (106). The patients, prior to the start of the 12-week trial, were metformin naïve and either received metformin (500 mg, twice each day) or placebo (106). Mather et al. reported that compared to placebo, metformin improved endothelium-dependent, but not endothelium-independent vasodilation thus highlighting the primary dysfunction in blood flow was not the vascular smooth muscle response but was secondary to endothelial dysfunction (106). A larger randomized placebo-controlled study with 390 patients receiving daily doses of between 850 and 2550 mg metformin focused on changes in biomarkers of endothelial dysfunction and patients were followed for 52 months (174). The results complement the plethysmography data reported by Mather et al. (106) with reduced levels of biomarkers of endothelial dysfunction including von Willebrand factor, soluble vascular adhesion molecule-1, tissue type plasminogen activator (t-PA), plasminogen activator inhibitor-1 (PAI-1), but not soluble E-selectin (sE-selectin), or urinary albumin (174). Data from a metabolomics analysis of T2DM patients treated with metformin has indicated that levels of the amino acid citrulline were lower than controls with comparable data from tissues from the db/db mouse model of T2DM and obesity. The metabolomics data can links the lowering of citrulline to the increase in NO generation from arginine and improved eNOS activity, thus providing support to the literature that metformin protects the cardiovascular system *via* promoting the generation of NO (175).

**Figure 2:**
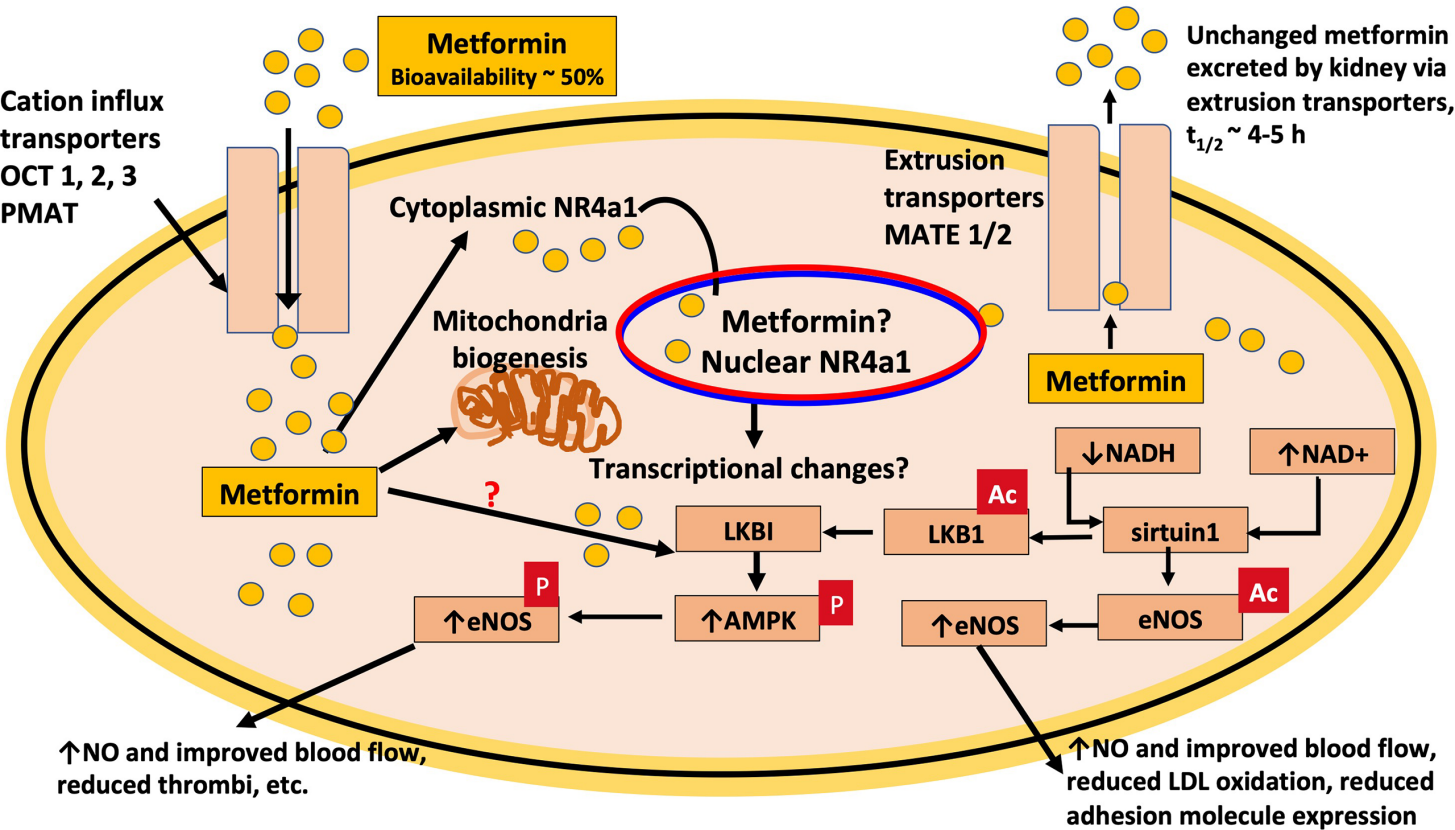
Metformin protects endothelial function. Pre-clinical and clinical data indicates that metformin has direct effects to protect the endothelium from diabetes-induced dysfunction and treatment results in improved function of endothelial nitric oxide synthase (eNOS), the generation of nitric oxide (NO) and improved blood flow that facilitates glucose disposal. Based on *in vitro* data, the effects of metformin are dependent on the expression of the NAD+ dependent deacetylase, sirtuin1, which targets lysine residues on eNOS as reflected in the figure by removal of ac, the activation of AMPK, and the nuclear orphan receptor, NR4A1. The latter has important links to the regulation of metabolism. OCT3 transporter expression in nuclear membrane facilitates metformin transport into the nucleus (183).

As previously discussed, metformin directly protects the endothelium from hyperglycemia-induced dysfunction and premature senescence and implicates a crucial role for the SIRT1-derived deacetylase, sirtuin-1 (110, 111). Sirtuin-1 plays an essential role in the regulation of angiogenesis, a protective role against oxidative stress and cardiovascular disease and higher expression levels of sirtuin-1 are associated with reducing disease (176–178). Sirtuin-1 also has been reported to promote endothelial cell proliferation and suppress senescence of porcine primary aortic endothelial cells *via* a signaling mechanism requiring the expression of the serine-threonine kinase, LKB1, the upstream regulator of AMPK (179). Sirtuin-1 plays a positive role in regulating endothelium-dependent vasodilation *via* the deacetylation of lysines 496 and 506 on endothelial nitric oxide synthase (eNOS) (180). The positive effects of metformin to offset hyperglycemia-induced endothelial dysfunction reflect an increase in the generation of NO (110, 111). In both studies, Arunachalam et al. (110) and Ghosh et al. (111) used 50 μM metformin and justified this concentration on the basis that estimates of metformin levels in the portal circulation following oral absorption are between 40 and 70 μM (4, 181). Of significance is that much lower concentrations of metformin, between 1 and 10 μM, have also been shown to protect endothelium-dependent vasodilation in murine aortae (182). In their study, Triggle et al. demonstrated a critical role for the orphan nuclear receptor, Nr4a1, in metformin’s endothelial-protective effects as the benefits of metformin were absent in orphan-nuclear-receptor Nr4a1-null murine aorta tissues (182). Furthermore, at the low micromolar concentrations used in this study, neither mitochondrial complex-I nor complex-III oxygen consumption rates were inhibited, but were when 500-μM metformin was used. Collectively, these data suggest that a novel mechanism of action is responsible for mediating the protective effects of metformin in the vasculature (**Figure 2**).

To unravel the precise signaling pathway linking metformin to the NR4A nuclear receptor family requires further studies, but it is worthy of note that the orphan receptor, Nur77, regulates LKB1 localization and is also important for the regulation of glucose uptake into C212 mouse muscle cells (184, 185). Additional evidence that metformin protects mitochondrial function has been provided by Wang et al., reported that treatment of streptozotocin (STZ)-induced diabetic mice with metformin (300 mg/kg/day) in their drinking water for 4 weeks suppressed mitochondrial fission through inhibition of dynamin-related protein (Drp1) *via* an AMPK-dependent process and enhanced mitochondrial fusion (186). Wang et al. have reported that metformin protects complex 1 activity in a cell culture protocol in hepatocytes but enhances mitochondria fission, thereby promoting healthy mitochondrial function *via* mitophagy (138).

Metformin and other drugs used for T2DM, like the SGLT2 inhibitors, may also reduce hyperglycemia-induced elevated endothelial ROS, independent of negative effects on complex 1, such as *via* inhibition of NADPH oxidase or blocking the entry of glucose into the endothelium (32, 107–109, 187, 188) (**Table 2**). Thus, based on both pre-clinical and clinical data, we can conclude that metformin elicits important protective effects on vascular function that help offset the advance of vascular-related diseases and thereby improves healthspan.

## Metformin and Hyperglycemic Memory

Hyperglycemic memory was first described in humans as the resistance to preventing the development of diabetic retinopathy despite achieving good glycemic control in patients (189). Hyperglycemic memory contributes to the pathophysiology of diabetes despite the initiation of intensive glycemic control (190, 191). Data from studies of tissues from streptozotocin-induced diabetic rats and also of endothelial cells in culture indicate that glucose-elevated fibronectin expression is not reversed when normal glycaemia is restored (192).

Metformin has been shown to reverse hyperglycemic memory. In bovine retinal capillary endothelial cells (BRECs) and retinas from diabetic rats, hyperglycemia-induced elevated levels of NF-κB, and Bax, a pro-apoptotic gene, were sustained even after returning to normoglycemia (193). BRECs where SIRT1 was knocked down with siRNA knockdown demonstrated an increased sensitivity to hyperglycemic stress, whereas SIRT1 overexpression, or exposure to metformin, inhibited the increase of mitochondrial ROS by upregulation of LKB1, and suppressed the expression of NF-B and the apoptosis regulator protein, Bax (193). Several other studies using a variety of different cell types have reported that metformin inhibits NF-κB activation, decreases the production of inflammatory cytokines and the genes that code for the inflammatory response thus supporting the healthspan benefits of metformin (105, 194–196).

## Exercise, Metformin, and Healthspan

Exercise activates AMPK, which in turn enhances glucose uptake into muscle and improves insulin sensitivity thus helping to offset the negative effects of obesity, diabetes and cardiovascular disease and thereby reducing morbidity and improving healthspan (197). It is important to note that exercise has been shown to have positive effects on the endothelium and, *via* improved eNOS function, enhances endothelium-dependent vasodilation. Metformin has also been reported to offset the effects of aging in the elderly and in cardiovascular disease (194, 198–200). On the other hand data from the Diabetes Prevention Program study of 3234 pre-diabetic subjects over an average of 2.8 years who were randomized to receive placebo, metformin (850 mg bid), or lifestyle intervention, indicated that although both metformin and lifestyle changes were effective in reducing the risk of developing diabetes, lifestyle intervention was more effective (7).

Since both exercise and metformin can improve glycemic control and since both mediate their effects *via* the activation of AMPK, this suggests that there should be at least an additive effect when metformin use is combined with exercise. Unfortunately, based on a prospective, double-blinded, randomized, controlled study additive effects of benefits were not observed (201). In this study, men and women with pre-diabetes followed an exercise protocol for 12 weeks with no drug, *versus* metformin alone (2000 mg/day), *versus* a combination, or, exercise plus placebo. The results indicated that although both metformin and exercise improved skeletal muscle insulin sensitivity by 55 and 90% respectively the combination resulted in only a 30% enhancement. The results were similar for effects on systolic BP and C-Reactive Protein (CRP) that were reduced by 7 to 8% *versus* 20-25% respectively (201). In addition, metformin blunted the exercise-induced increase in VO_2peak_ (201). The authors suggest that the negative effect of metformin on exercise results from metformin lowering ROS levels, thus reducing the effects of ROS to activate AMPK. The data suggest that exercise, and not metformin, is the *“ideal drug”* (201).

Additional doubts about the benefits of combining metformin and exercise come from two studies with older adults. Konopka et al. reported that metformin (2000, or 1500 mg/day for those experiencing GI upset) attenuated exercise induced increases in whole-body insulin sensitivity and also reduced exercise-induced increases in mitochondrial respiration in patients with family history or risk factors for T2DM (202). In the double-blinded Metformin to Augment Strength Training Effective Response in Seniors (MASTERS) trial (203), metformin, despite an increase in AMPK signaling, blunted the exercise-induced hypertrophic response in skeletal muscle in healthy men and women over the age of 65 who took part in a supervised progressive resistance exercise training program over 14 weeks following a 2 week metformin treatment (1700 mg/day, or placebo) (203). Comparable conclusions were reached based on the data from the Look AHEAD randomized intensive lifestyle intervention trial that metformin provided minimal additional benefit (204). Exercise is universally endorsed as the ‘Gold Standard’ for improving cardio-respiratory health and analysis of the benefits of exercise for 26 different chronic diseases reflects positively on *“Exercise as Medicine”* (205). But the negative effects of metformin on exercise-induced benefits raise concerns about the use of metformin for anything other than for approved diseases such as T2DM.

## Calorie Restriction and Nutrient Signaling Pathways

**Figure 3:**
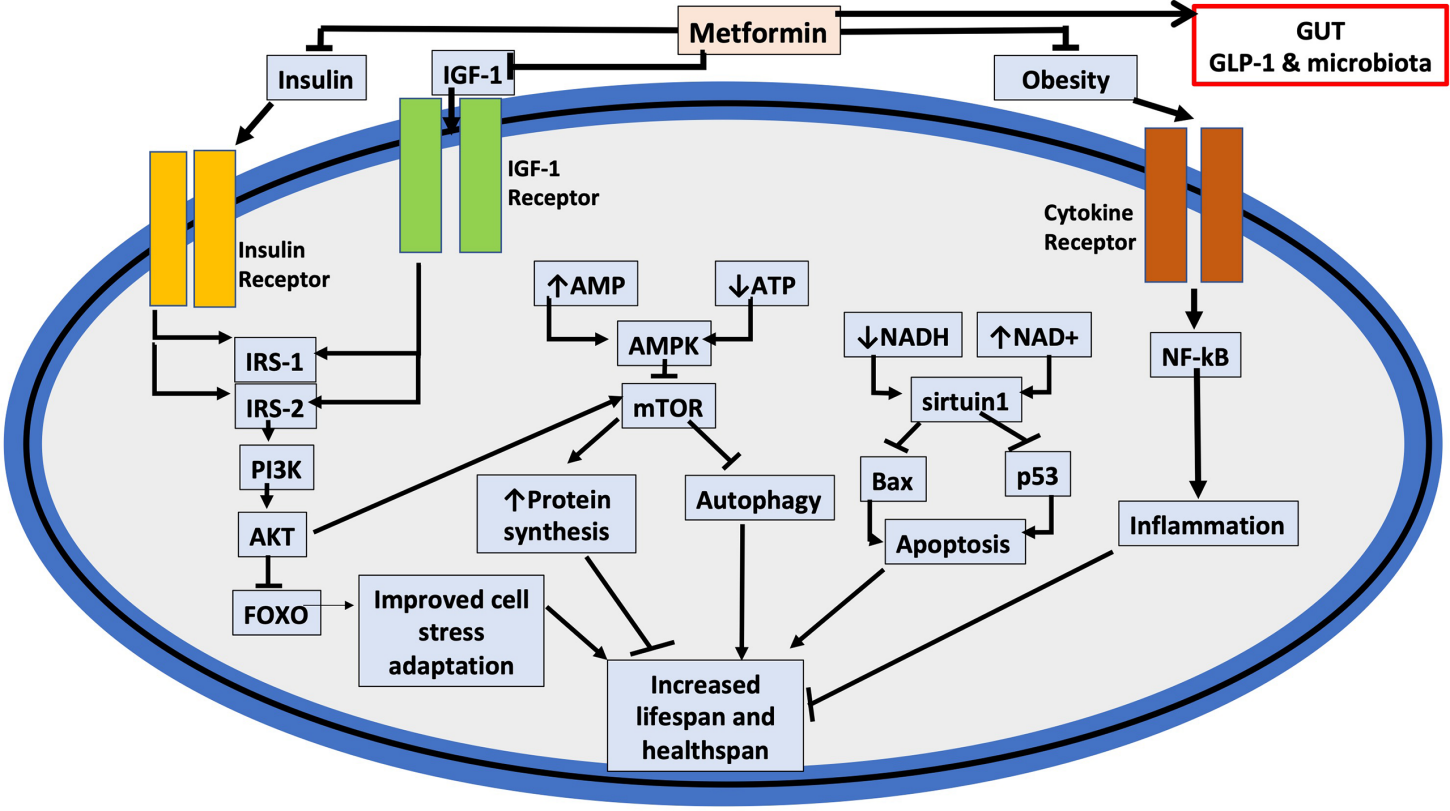
Potential cellular targets for metformin that affect healthspan and lifespan. The figure depicts how metformin may affect cell aging and indicates a potential action in the gut where, prior to absorption, merformin modulates the microbiome as well as enhances release of glucagon-like factor 1 (GLP-1). Important links are also indicated to the insulin (IRS: Insulin Receptor Substrate) and insulin-like growth factor-1 (IGF-1) signaling pathways as well as to tumor suppressors including p53, and inflammation and cytokine signaling. (PI3K: Phosphatidylinositol 3-kinase); (AKT: protein kinase B); (FOXO: Forkhead Box O3); (SIRT1: NAD-dependent deacetylase sirtuin-1); (Bax: Bcl-2-associated X protein). As a result of metformin moderating the cellular signaling pathways mediated by insulin, IGF-1, and cytokines, both, healthspan and lifespan are increased. Metformin also, inhibits the inflammatory pathway and increases AMPK activation, which inhibits mTOR, a primary target for cell aging modulation. Inflammation, apoptosis, autophagy, cell survival, and protein synthesis are all affected by these mechanisms and are all linked to accelerated aging.

Several genetic mutations, such as in the *C. elegans* DAF-16 transcription signaling pathway, involving nutrient-sensing pathways have been described and linked to a role in extending lifespan. The data infer the mutations cause a physiological state similar to that experienced during periods of reduced calorie intake (206). Caloric restriction of 10-15% without malnutrition has been suggested to have a role in increasing lifespan in humans (70) (see **Table 1**). Furthermore, caloric restriction has been found to extend the life span of several organisms including S. cerevisiae (yeast), C. elegans, fish, rodents, and rhesus monkeys (206, 207). Caloric restriction reduces the generation of growth hormone, insulin, IGF1, and other growth factors, all of which have been shown to hasten aging and increase mortality in a number of species (see **Figure 3**) (206).

A longitudinal study suggested that long-term calorie restriction of 30% significantly reduces age-related deaths in adult rhesus monkeys and a 50% lower incidence of cancer and cardiovascular disease as compared to control animals (208). Apart from lifespan extension, caloric restriction also reduces the risk factors for major diseases including diabetes and cardiovascular disease in rodents (209, 210). Studies by the Calorie Restriction Society, a group of people who chose to limit their calorie consumption with the intention of extending their life span, included adult men and women (mean BMI, 19.6; mean age, 51 years; age range, 35-82 years) whose diet of nutrient-dense foods, consisted of approximately 1800 kcal/day for an average of 6.5 years and consumed 30% fewer calories than age and sex-matched adults on a standard Western diet (210–212). Those on the calorie-restricted diet demonstrated a number of metabolic improvements including body fat, lower blood pressure, and improved insulin sensitivity, and lipid profile (210–212). Meta-analysis has also demonstrated that dietary restrictions decreased levels of circulating IGF-1 in humans (213); however, although much of the evidence indicates that lower levels of IGF-1 benefit enhanced lifespan, IGF-1 does play important roles in homeostasis and not just during childhood thus raising the concern of potential negative effects of excessive lowering of the growth hormone.

Collectively, these observations support the benefits of calorie restriction and heighten the interest in pharmacologic agents, such as metformin, as calorie restriction mimetics; however, there are questions and limitations to address that include [see (214, 215)]: For instance: 1. What level of calorie restriction is required and is acceptable for optimal benefit? 2. How to avoid the effects of severe calorie restriction that can result in malnutrition and adversely affect health particularly in those with low BMI? (214, 216). These questions are important as not all studies have demonstrated a clear relationship between BMI, being overweight, obese and mortality although the risk of CVD is elevated (217). Dietary intervention studies such as with the Women’s Health Initiative RCT have also generated contradictory results with respect to CVD risk (218).

The cellular processes that mediate the benefits of calorie restriction on lifespan in mammalian species remain controversial and although it is tempting to assume an important role for AMPK as a key nutrient sensor, there are a number of caveats that limit a positive correlation in mammalian species *versus* more convincing evidence in lower eukaryotic organisms, such as *C. elegans* (219). For instance, as discussed in the next section, despite activating AMPK, metformin has not reproducibly been shown to enhance lifespan in rodents and notably less, or not effective in older animals, including *C. elegans* (66, 72, 74, 75, 77, 78). In addition, applying data obtained from studies with *C. elegans* and rodents to intervention studies in a diverse human population raises obvious limitations.

## Calorie Restriction Mimetics

The National Institute on Aging Interventions Testing Program has investigated the effectiveness of a variety of pharmacologic agents, including aspirin, metformin, nordihydroguaiaretic acid (NDGA), and rapamycin to determine whether they prolong lifespan in mice (220). Of significance is that metformin, like rapamycin, is known to inhibit mTOR signaling, and the inhibition of the mTOR signaling pathway with rapamycin has been shown to extend lifespan in *C. elegans*, S. cerevisiae, and Drosophila melanogaster (fruit fly) (221–225). The lifespan-extending effects of metformin have been investigated by Martin-Montalvo et al. in male mice, and indicate that long-term treatment with 0.1 percent metformin w/w supplemented in the diet and beginning in middle age, increased healthspan and lifespan (76) (see **Table 1**). A higher dose of 1% metformin w/w, on the other hand, was toxic and decreased the average lifespan of mice by 14.4% (76). The effects of metformin were reported to be similar to those of calorie restriction, including improved insulin sensitivity and lower cholesterol levels (76). However, Strong et al. were unable to reproduce the positive lifespan data with the 0.1% metformin protocol in mice, although in combination with rapamycin (14 ppm) lifespan was extended (77). Strong et al. provided the explanation that the insulin-sensitizing effect of metformin offsets the negative effects of rapamycin on glucose homeostasis (77). Smith et al. have demonstrated that calorie restriction, but not metformin (in the diet 300 mg/kg/day), extended lifespan in male Fisher-344 rats, leading to the conclusion that metformin is not a calorie restriction mimetic (CRM) (78) (see **Table 1** for a summary).

Collectively, these results demonstrated that the mTOR pathway has a role in lifespan extension in mammals, but raise the question as to why, with the exception of the study by Martin-Montalvo et al., metformin does not extend lifespan in mammals whereas rapamycin does (76). Blagosklonny argued that the metabolic side effects of rapamycin are a consequence of it acting as a CRM and are required to mediate its positive effects on lifespan (226). If we accept this argument and also accept that metformin, despite inhibiting mTOR, is not a CRM (77, 78) then metformin would not be expected to extend lifespan. One caveat to consider for all ageing studies using rodent models relates to the marked genomic differences between rodents and humans in terms of the response to inflammatory disease (227). No doubt, both the innate and adaptive immune responses to inflammation play a key role in the ageing process, and differences in these responses between rodents and humans merit attention. In this regard, metformin may play an important role, due to its potential impact on the innate immune response and the generation of ROS caused by inflammatory cytokines.

## Metformin and Autophagy

Autophagy is a process necessary for the removal of damaged proteins and organelles and plays an important role in the regulation of cell aging, providing a supply of nutrients to maintain cellular function during starvation, and inhibition of autophagy mimics accelerated aging (52, 228, 229). Furthermore, calorie restriction is a strong inducer of autophagy and increases the lifespan in *C. elegans* (230).

Xie et al. investigated the role of chronic AMPK activation by metformin in restoring cardiomyocyte autophagy in OVE26 diabetic mice, a model for type one diabetes (231). Isolated hearts, from the diabetic mice showed a substantial reduction in AMPK function and cardiomyocyte autophagy as well as mitochondria aggregations dispersed between poorly organized myofibrils and increased apoptosis that was reversed following chronic treatment with metformin (231). Song et al. have reported a link between SIRT1, AMPK, and metformin-induced autophagy thereby supporting a synergistic relationship between the deacetylase, sirtuin-1, and metformin-mediated effects on aging (232).

In mice, overexpression of Atg5, the protein product of the essential gene for the autophagosome, boosts autophagy and, more importantly, induces anti-aging phenotypes including enhanced insulin sensitivity and motor control (233). Additionally, embryonic fibroblasts cultured from Atg5 transgenic mice are less affected by oxidative stress-induced cell death, a tolerance reversible by an autophagy inhibitor (233). Furthermore, siRNA targeting Atg5 blocks metformin’s activation of autophagy flux and cell death in adenocarcinoma cells in culture, albeit based on the use of high concentrations (1 to 4 mM and a 12-hour exposure) of metformin (234).

Collectively, data from these studies suggest a link between metformin, autophagy, and extension of lifespan. However, under conditions when tumor microvascular endothelial cells in culture are exposed to glucose starvation, metformin inhibits autophagy *via* inhibition of the mTOR pathway and a partially AMPK-independent mechanism in (235). The effects of metformin to inhibit autophagy were only seen following a 48-hour incubation with 2 mM metformin, and lower concentrations that are in the therapeutic range, including 50 μM, were ineffective (235). Thus, again the question is raised as to whether effects reported from *in vitro* studies with mM concentrations of metformin can be translated to a therapeutic effect in humans.

## Metformin and a Decreased Incidence of Cancer

Diabetes has been associated with an enhanced risk for the development of various cancers (236). A retrospective study published in 2005 reported that patients with diabetes who had been treated with metformin for T2DM had a lower risk of cancer and highlighted the possible link between metformin and the serine-threonine tumor suppressor, LKB1, as a mechanism for the reduced risk (113) (see **Table 2**). Similarly, the link between metformin and the activation of AMPK has been emphasized as the basis for the anti-proliferative effects of metformin (43).

Extensive support for a protective effect of metformin against cancer has been provided by numerous, but not all studies (237, 238). For instance, no association has been shown between the use of metformin and a lower incidence of bladder cancer and concerns have also been expressed how data from observational studies are analyzed (239, 240). The National Cancer Institute (https://www.cancer.gov/about-cancer/treatment/clinical-trials/intervention/metformin-hydrochloride) lists a number of on-going clinical trials involving metformin for the following: Her2 positive breast cancer, head and neck squamous cell cancer, endometrial and ovarian cancers, multiple myeloma, lymphocytic leukemia, and thyroid cancer. Examples include a Phase II study, NCT02028221, designed to determine whether metformin reduces obesity-associated breast cancer risk and due to be completed in mid-2021. A Phase III trial, NCT01101438, *“Metformin Versus Placebo on Recurrence and Survival in Early Stage Breast Cancer”* is due to be completed in early 2022.

A logical target whereby metformin could mediate its putative antiproliferative effects in cancer is *via* inhibition of mTOR and the serine-threonine kinase, ribosomal S6K (pS6K), either *via* activation of AMPK or *via* an AMPK-independent pathway (241, 242). It has also been argued that the inhibition of mitochondrial complex 1 is an important contributor to the cytotoxic effects of metformin and has been observed in cancer cells and supported by data showing reduced inhibition of tumor growth in cancer cells expressing a metformin-resistant yeast complex 1, NDI1 (136, 243).

The metabolic changes that occur as a result of diabetes (hyperinsulinemia, hyperglycemia, and dyslipidemia) potentiate signaling pathways and may increase the oncogenic nature of breast tissue through accelerating cell growth and migration, angiogenesis, increasing metastasis, and decreasing the response to chemotherapy (158, 244–247). These metabolic pathways, rather than a direct anti-proliferative action, may be the target as Metformin decreases hepatic gluconeogenesis, improves insulin sensitivity, reduces insulin and blood glucose levels, and these effects, which also will reduce tumor growth, rather than a direct anti-proliferative action may be the primary target of metformin (55, 248).

As previously stated when used to treat diabetes peak plasma concentrations of metformin are usually less than 20 μM (155, 160). However, many *in vitro* studies have used mM concentrations of metformin to demonstrate an anti-proliferative action in tumor cells in culture [see (114, 115); **Table 2**]. Chandel et al. argue that higher concentrations of metformin are necessary in *in vitro* cell culture protocols because the abundance of growth factors and nutrients, such as glucose, reduces the sensitivity to the inhibitory effects of metformin thus reflecting the importance of glucose and the Warburg effect for cancer cell growth as well as the importance of glycemic control in diabetes (249). Studies of the effects of glucose concentration on the anti-proliferative effects of metformin on breast cancer cell growth in different human cancer lines *In vitro* studies reveal that triple negative breast cancer cells (TNBC) are particularly sensitive to the pro-proliferative effects of glucose, and TNBC cells are more sensitive to metformin at lower levels of glucose (250). Zordoky et al. reported that metformin significantly inhibited growth in cells cultured in normoglycemic conditions, and only for the cells grown in normoglycemic conditions did metformin induce significant AMPK activation (251). Samuel et al. have also demonstrated using a cell culture protocol that higher levels of glucose reduce the ability of metformin to inhibit cancer cell proliferation (247). In contrast to the data from *in vitro* studies, a retrospective analysis of patients with T2DM and TNBC by Bayraktar et al. indicated that treatment with adjuvant metformin was not associated with a significantly improved survival and concluding the need for data from prospective Phase III randomized studies (252).

An argument to explain the selective action of metformin in some cancers but not all is that there is a differential expression of the influx and efflux transporters in tumor cells that allows for the intracellular accumulation of metformin in the cancer cell and resultant selective toxicity (158). Cai et al. who compared metformin uptake levels and inhibiting activity on cancer cell growth in a human breast cancer cell line (BT-20) deficient in OCTs and a BT-20 cell line overexpressing organic cation transporter 3 (OCT3), OCT3-BH20 cells: OCT3 is also a predominant transporter in human breast neoplasms (241). [^14^C]-Metformin uptake was 13 times higher in OCT3-BT20 cells than in BT-20 cells, and associated with increased AMPK phosphorylation and decreased pS6K phosphorylation in OCT3-BT20 cells and these results were substantiated in mice with OCT3 overexpressing tumors (241). Comparable data has also been reported from a study with a high-fat diet rat model of breast cancer showing a positive association with the expression of OCT2 and accumulation of [D_6_]-metformin isotope (253). In contrast and although LnCaP, a prostate cancer line, proved to be particularly sensitive to metformin and in a concentration range that was within that expected clinically and correlated with a high expression of mRNA for OCT3 and low expression of MATE2 for the other cell lines, very high concentrations, up to 10mM, of metformin were required to see significant inhibition, and a strong significant correlation between inhibition of proliferation and MATE2 expression was not seen (254). A limitation of the studies investigating expression levels of the cation transporters is that in the absence of adequate specific transporter antibodies quantification of transporter protein was not possible, and correlations were based entirely on mRNA data. Collectively, these data suggest that expression levels of the uptake and extrusion transporters are not necessarily the limiting factors determining the anti-proliferative effects of metformin and that genetically determined variations in signaling pathways are variably important as are nutrient levels in the tumor microenvironment (249, 254).

In conclusion, since beneficial effects of metformin are not seen in all cancers further studies are required to determine whether the anti-cancer actions are direct or are secondary to the positive effects of metformin on healthspan that are apparent in obese patients such as improved glucose homeostasis, enhanced insulin sensitivity and reduced signaling through the IGF-1–mTOR pathway (114, 242), **Table 2**.

## The Connection Between Metformin and Improved Neurological Function

Diabetes-associated hyperglycemia, hyperinsulinemia, elevated oxidative stress, vascular disease, and inflammation are all linked to cognitive decline and, as reflected by a meta-analysis, it was concluded that metformin reduces cognitive decline and dementia in T2DM subjects (255).

In the Singapore Longitudinal Aging Study 2,365 subjects with diabetes (age ≥ 55) were monitored for 4 years – see **Table 2** (116). In a cross-sectional and longitudinal multivariate analysis, odds ratios of association of the chronic use of metformin with cognitive disability (Mini-Mental State Exam ≤ 23) were evaluated demonstrating a substantial inverse relationship with cognitive impairment (116). In a large observational study, 67,731 participants, who had no evidence of dementia, were non-diabetic, and aged ≥ 65 were followed from January 2004 to December 2009, to observe the onset of T2DM and compare the risk of the incidence of dementia associated with the use of anti-diabetic drugs and reported that metformin reduced the risk of developing dementia (256). In another clinical trial, 58 participants who had both depression and T2DM received either metformin or placebo for 24 weeks concluded that metformin improved cognitive performance (257).

A molecular basis for the effects of metformin on cognitive function is suggested by data using adult murine neural stem cells in culture that shows metformin in the concentration range 500 nM to 1 μM enhanced proliferation and self renewal dependent on the transcription factor, Tap73, and enhanced neuronal differentiation *via* the AMPK-atypical protein kinase C (aPKC)-CREB-binding protein (CBP) pathway [(117): **Table 2**]. Data from a study with high-fat diet mice has also linked the learning and memory restorative benefits of metformin in the diet (250 mg/kg/day) to the microbiota with positive data from a fecal transplantation protocol [(118): **Table 2**]. The potential to use metformin to treat Alzheimer’s is also provided by data indicating that metformin targets monoacylglycerol lipase, an enzyme responsible for generating pro-inflammatory eicosanoids from 2-arachidonoyl glycerol (258). An overlooked hypothesis is that the ability of metformin to enhance CNS vascular function, as it does in the periphery (106), may also contribute to improved CNS function.

## Clinical Trials to Assess Effects of Metformin on Aging, Healthspan, and Lifespan

Designed as a crossover study, the *Metformin in Longevity Study* (MILES) is a double-blinded study where the subjects act as their own placebo control group [(79) **Table 1**]. MILES (https://clinicaltrials.gov/ct2/show/NCT02432287) commenced October 2014 and was conducted on 14 elderly participants with impaired glucose tolerance to determine whether metformin (1700 mg/day) can cause physiological and transcriptomic changes in muscle and adipose tissues after 6 weeks of treatment and also to determine which pathways are affected by metformin and outline possible molecular intermediates involved in metformin’s mechanism of action (79). Data from the MILES trial indicate that metformin modified multiple pathways associated with aging including metabolic pathways, collagen trimerization and extracellular matrix (ECM) remodeling, adipose tissue and fatty acid metabolism, mitochondria, and the MutS genes, MSH2 and MSH3, which play a role in DNA mismatch repair, a process that declines with age (79).

Dysregulation of adipose tissue metabolism has been previously linked to an age‐related process of ECM deposition and the effects of metformin on fatty acid metabolism have been previously shown to mimic those of other interventions that increase lifespan in model organisms (36, 259, 260). The results of the MILES study underscore metformin’s targeting of multiple mechanisms of aging. However, it is important to note a concluding sentence from Kulkarni et al.: “*These findings remain to be validated in other tissues and study designs and do not yet allow us to identify the primary site of action of metformin, which then may trigger the observed changes in gene expression”* (79).

Targeting Aging with Metformin (TAME) trial is a double-blinded, placebo-controlled, multicenter trial that is planned to involve 14 research centers in the USA, and subject to funding and approval, will enroll 3000 ethnically diverse, non-diabetic subjects aged 65–80 (60, 261). The objectives of TAME are: 1. Clinical outcomes as measured by the appearance of new age-related chronic diseases; 2. Functional outcomes such as changes in mobility as measured by gait speed over 10 meters [also see (262)], as well as measures of cognitive impairment; 3. Biomarkers of aging such as for inflammation and senescence (261). The study plan for TAME is that patients will be given a daily dose of metformin (1500 mg) for 6 years, with an estimated follow-up period of more than 3.5 years (261). It is argued that TAME trial outcomes will give more insight on whether metformin decreases the risk of developing age-dependent diseases, excluding diabetes, in non-diabetic individuals, and potentially provide a tool to target aging itself and not related diseases individually (60, 261). However, as expressed both in this review and by others there are concerns about the age –dependent effects of metformin and in older organisms, including C.elegans, rodents and humans, the effects of metformin are variable and may be detrimental [see (202)]. It is also worthy of citing a concluding statement and caution by Pyrkov et al. from their dataset analysis of more than 500,000 people from Russia, UK and USA to assess biological age and the potential for extending lifespan: *“The proximity of the critical point revealed in this work indicates that the apparent human lifespan limit is not likely to be improved by therapies aimed against specific chronic diseases or frailty syndrome.”* (263).

A number of other clinical trials are underway that address some of the concerns noted in this review: NCT04264897 (*Does Insulin Sensitivity Impact the Potential of Metformin to Slow Aging*) due to be completed in April 2024, which is a randomized, placebo driven, double-blinded Phase 3 study with 148 participants and the objective to compare the effects of metformin on insulin sensitivity and mitochondria function in patients who are insulin-sensitive *versus* those who are insulin resistant. NCT02570672 (*‘Metformin for Preventing Frailty in High-risk Older Adults’*) is a Phase 2 study involving 120 subjects aged 65 to 90 years old with pre-diabetes and treated with 1000 mg metformin bid, *versus* placebo, and due for completion in October 2024. Frailty will be determined using a standardized assessment (264). A Phase 1 study already underway, *Role of Metformin on Muscle Health of Older Adults* ‘(NCT03107884), and due for completion in April 2022 is designed to determine whether metformin treatment of elderly subjects offsets the negative effects of bed rest on lipid accumulation, inflammation, insulin resistance, and muscle loss. Collectively the results from these studies will be valuable in better assessing the benefits of metformin on healthspan and also fine-tuning future larger studies such as TAME.

## Conclusions

Based on a 60-year history of use as an anti-diabetic drug for the treatment of T2DM, metformin is accepted as a comparatively safe drug. Metformin is no longer protected by patents and thus is comparatively inexpensive. Collectively, these attributes together with an extensive literature supportive of benefits in the settings of diabetes, obesity, cardiovascular disease and, arguably, cancer and dementia could justify its wider use as a prophylactic to offset the effects of aging and enhance healthspan and lifespan. In this review we have also highlighted and critiqued some of the key clinical and laboratory-based studies that provide data supportive of the hypothesis that metformin, independent of its anti-hyperglycemic actions, has benefits that in principle can slow cellular aging and enhance healthspan and lifespan. Metformin, *via* its direct protective effects on vascular function, may slow the aging process *via* improved blood flow and provide protection against age-related cognitive decline. However, not all of the data is supportive and metformin, as shown in C. elegans and mice, may be less effective, or ineffective, in older humans. We have also stressed that, based on the pharmacokinetic properties of metformin, caution is needed before extrapolating from *in vitro* cell-based studies done with comparatively high metformin concentrations to clinical effectiveness with plasma concentrations in the range of 20 micromolar or lower. This issue is a particular concern with the *in vitro* studies reporting an anti-proliferative effect of metformin and promoting its use as an adjunct for the treatment of various cancers as, in most of these studies, the concentration/dose of metformin that have been used would most likely prove toxic in patients. Furthermore, a dependence on the use of metformin as a prophylactic to delay aging could serve to decrease the incentive to pursue the proven benefits of lifestyle changes such as improved diet and exercise. Moreover, the long-term chronic use of metformin would require attention to the potential occurrence of vitamin B12 deficiency. On this basis, we conclude that metformin should not be seen as a ‘quick fix’ panacea for aging at the expense of non-pharmacologic interventions such as diet, exercise, and related lifestyle changes. Indeed, the use of metformin may negate some of the positive effects of exercise and lifestyle and less favorable effects in older subjects as was also emphasized by the Diabetes Prevention Program (265, 266). On the more positive side, we do accept that the use of metformin in the treatment of patients with T2DM is associated with a positive benefit on healthspan. By lowering plasma glucose levels and body weight, metformin improves the metabolic profile of the patient and thereby reduces the severity and risk of other diseases associated with diabetes such as cardiovascular, cancer, and also neurodegenerative diseases (267). The importance of the gut-brain axis in mediating the therapeutic effects of metformin is also emphasized, as are the potential beneficial effects of metformin to protect against neurodegenerative disorders. Finally, although the evidence for lifespan expansion in mammalian species is not conclusive, a full analysis and follow-up of clinical trials, including MILES and TAME, may provide more definitive answers as to whether metformin should be promoted beyond its use to treat T2DM, as a drug that enhances both healthspan and lifespan. Of particular importance is the need for evidence from prospective studies of the effects of metformin on subjects of different age groups, free of chronic diseases, which will help determine if metformin has benefits beyond those of reducing pre-existing disease burden.

## Author Contributions

IM and CT participated in the manuscript conceptualization and writing the first draft. HD, MH, IM, and CT contributed equally to literature and manuscript review and revisions of the manuscript. All authors contributed to the article and approved the submitted version.

## Funding

The publication of this article was funded by the Department of Medical Education, Weill Cornell Medicine.

## Conflict of Interest

The authors declare that the research was conducted in the absence of any commercial or financial relationships that could be construed as a potential conflict of interest.

## Publisher’s Note

All claims expressed in this article are solely those of the authors and do not necessarily represent those of their affiliated organizations, or those of the publisher, the editors and the reviewers. Any product that may be evaluated in this article, or claim that may be made by its manufacturer, is not guaranteed or endorsed by the publisher.
